# Embodiment and personal identity in dementia

**DOI:** 10.1007/s11019-020-09973-0

**Published:** 2020-08-31

**Authors:** Thomas Fuchs

**Affiliations:** Karl-Jaspers-Professor of Philosophical Foundations of Psychiatry, Psychiatrische Universitätsklinik, Voss-Str. 4, 69115 Heidelberg, Germany

**Keywords:** Dementia, Personal identity, Embodiment, Body memory, Pre-reflective self-awareness, Reflective self-consciousness

## Abstract

Theories of personal identity in the tradition of John Locke and Derek Parfit emphasize the importance of psychological continuity and the abilities to think, to remember and to make rational choices as a basic criterion for personhood. As a consequence, persons with severe dementia are threatened to lose the status of persons. Such concepts, however, are situated within a dualistic framework, in which the body is regarded as a mere vehicle of the person, or a carrier of the brain as the organ of mental faculties. Based on the phenomenology of embodiment, this paper elaborates a different approach to personal identity in dementia. In this perspective, selfhood is primarily constituted by pre-reflective self-awareness and the body memory of an individual, which consists in the embodiment and enactment of familiar habits, practices and preferences. After describing the different types of body memory, the paper develops a phenomenology of dementia as a loss of reflexivity and meta-perspective. This is contrasted with the preservation of individual forms of body memory even in the later stages of the illness. The ethical consequences of an embodied approach to dementia are outlined. A final look is given to narrativistic and constructionist concepts of the self in dementia.

## Introduction

“I have, so to speak, lost myself”—this was the complaint of Auguste Deter, the first patient who was diagnosed by Alois Alzheimer in 1901 with the illness that was later named after him (Maurer et al. 1997). Alzheimer’s disease and other dementias seem particularly unsettling and threatening, as they call into question that which we perceive as the foundation of being ourselves: our cognitive and reflective capacities. To be a person in the full sense of the word is, in Western cultures, decisively bound up with the intactness of functions such as consideration, rationality, memory, and with the autonomy that is based on them. Impairments resulting from a process of dementia therefore come into conflict with the central values of a culture centered on cognition and on the individual.^1^ Dementia becomes a threat to the person as such and is more stigmatized than most other mental illnesses: in the advanced stages, the loss of reason and autobiographical memory appears solely to leave behind a bodily facade, the utterances of which allow only the recognition of fragments of the earlier person. For ethical utilitarians such as Singer (1979) or McMahan, people with severe dementia appear consequentially no longer to be persons as such, but rather as “quasi-persons” or “post-persons” (McMahan 2003, 46 ff., 55).^2^

However, this identification of our selfhood with cognition, rationality, and memory is based, on the one hand, on a dualistic conception of personhood, in which the body serves only as a vehicle for the mind—or the brain, respectively. According to this view, the cortex and the act of thinking become the site of the person, while the rest of the body as well as embodied feelings—without cognitive awareness and rational control—lead nothing more than a shadow existence. On the other hand, such a view neglects what is constitutive of human personhood, namely its sociality, which already manifests itself in the primary, pre-reflective intersubjectivity of early childhood, and which is crucially based on intercorporeality and interaffectivity (Stern 1985; Trevarthen 1993; Fuchs 2017a).

In this paper, I will oppose the cognitivist perspective on dementia to another conception of personhood, which has its foundation in the phenomenology of the body. Here, our primary selfhood is essentially vital and bodily. Only as a body can a human sense and express itself, and encounter other humans and the world. Everything to do with perception, thought, and action is performed through the medium of the body: the eyes see, the ears hear, the hands hold and the tongue speaks, all without our direct awareness. Whatever we consciously plan or do, we proceed from a bodily foundation which we are never able to make fully conscious to ourselves. This foundation, as I am going to show, is never completely lost even in dementia. Even more, it is also the basis of an intercorporeality that always already connects us with others, without requiring explicit, symbolically mediated interaction.

The subjective or lived body also has its own history. From early childhood, its experiences have sedimented as sensorimotor habits and capabilities of dealing with objects and other people. All of these habits and experiences can be brought together in the term *body memory*. This points towards a continuity of the person which is not rooted in in a repertoire of memories, but rather in experience sedimented in the body. It is only recently that this form of memory has been taken into account for understanding and treating dementia.^3^ And, indeed, it is a kind of memory which remains preserved right up to the last stages of the illness, and in which the biographical history of the patient is manifested.

In what follows I will first consider personal identity from a cognitivist and from an embodied perspective, then focus on the role of body memory for the continuity of the self. Turning to dementia, I will first describe it as a loss of reflexivity and meta-perspective, which I will then contrast with the preservation of body memory and intercorporeality. This will allow to argue for the continuity of the pre-reflective self even in late stages of the illness. A final look will be given to narrativistic and constructionist concepts of the self in dementia which I contrast with an embodied notion of personhood.

## Personal identity

The cognitivist conception of person has a history which goes back to the origins of European modernity and which is characterized by the growing separation of the personal subject from its corporality and vitality. The dualistic and rationalistic understanding of person in the work of Descartes or Locke is bound up with self-consciousness, consideration and rational reflection, as the modern subject wants to be certain of itself, sovereign and autonomous. However, this certitude of *cogito* is only ever possible as an instantaneous self-consciousness. No longer embedded in its corporeality, the Ego must continuously think in order to exist, and reflect upon itself, in order to be certain of itself. Yet what does the *res cogitans*, that thinking thing, do when it does not think, when it has to surrender to the body, to sleep or forgetting? What is it then that enables the lasting continuity of the person? Early on, Locke identified this as a problem with the Cartesian subject:

But that which seems to make the difficulty, is this, that this consciousness being interrupted always by forgetfulness (…) in all these cases (…) – doubts are raised whether we are the same thinking thing; i.e. the same substance or no (Locke, Essay II, xxvii; cf. Locke 1997, 303).

Locke’s solution, which has been influential until today, was the following: it is self-consciousness and memory which allows a person to extend herself into time beyond the present:

For as far as any intelligent being can repeat the idea of any past action, and with the same consciousness it has of any present action; so far it is the same *personal self* (Locke 1997, 303).

Memory thus builds the necessary bridge: the unity and identity of the person is bound to the possibility of conscious remembering. With its help, past episodes are assimilated and integrated in the present self. This means, however, that I remain myself only so long as I can recall my previous states and accredit them to myself. Locke’s view continues to this day in the psychological conception of personal persistence^4^: the identity of a person reaches only as far as his memory of himself remains intact. Yet, this view has the counterintuitive consequence that we can strictly speaking neither ascribe states of sleep nor our fetal and infant states to ourselves, for these are not states we can remember. Moreover, if a patient in schizophrenia is delusional and considers himself another person, he would lose his personhood for that period of time. The same would apply to a patient with advanced dementia who can no longer remember his or her previous experiences.

But in which way is the self lost in dementia? Is it really the case that our selfhood, our identity, depends solely on our memory and knowledge of ourselves? By no means, for this knowledge of the “self-as-object” is preceded by the “self-as-subject”, a continual, pre-reflective experience of self which does not need to be made explicit or grasped in words. Most of the day, we do not make ourselves conscious of who we are, do not reflect on ourselves or recall autobiographical memories—we are just aware of ourselves as a matter of course (Klein 2013; Fuchs 2017b). There is no need for being reflectively self-conscious, remembering the past, or other forms of explicit psychological connectedness which Lockeans have in mind. Moreover, in our first awakening from sleep, we already find ourselves in our basic bodily sense of self, prior to any memory of the day before, even less of our biography.^5^ Reflective self-consciousness is a possibility, but not a necessary condition of being oneself. We could persist as selves with pre-reflective self-awareness, based on the background feeling of the body, even though this is normally superimposed by multiple facets of self-reflection, self-knowledge, and memories, or in other words, by the self-as-object. So when Auguste Deter complained “I have lost myself”, she obviously still *had* a sense of self, otherwise her sentence would not have had a subject. What she had lost was the *self-as-object*, or her knowledge about herself, not the self-as-subject.

As Locke did not distinguish between reflective and pre-reflective self-experience, he could only base the sense of identity on recollection. However, the lived body conveys a continuity of selfhood, which ultimately represents the subjective side of the life process itself and does not require reflective self-identification. In this sense, Merleau-Ponty described the lived body as the “natural subject”, which is the precedent and foundation for all conscious and reflective acts.^6^ Were we to be without this basal experience of self, then all biographical knowledge would be useless to us, as our self would be lost in an elementary sense, and there would be no one to ascribe this knowledge to.^7^ As meaningful the possible grasp of this knowledge might be for our *narrative* identity, selfhood in a foundational sense is not bound to biographical memory nor to knowledge about oneself. It is rather an intrinsic quality of every experience, a self-givenness of the continuous stream of consciousness as such (Zahavi 1999, 2006). As we will see, such a basal self-experience remains intact even in late stages of dementia.

The phenomenology of the bodily subject can be expanded to a conception of *embodied personhood* which integrates the aspect of the lived body (*Leib*) and the aspect of the living body or organism (*Körper*). This can only be outlined briefly here (for an extensive depiction, see Fuchs 2017b, 2018): According to the paradigm of embodied cognition, consciousness is not a pure product of the brain, but is rather a comprehensive activity of the entire organism in relation to its environment. Only a brain connected to a sensory, perceptive and moveable body is in a position to serve as an operating organ for psychological processes; the reason for this is that it is only through the continuous interaction between brain, body, and environment that the forms of conscious experience emerge and stabilize themselves. In this respect, personhood is a manifestation of the life process of a human organism and it is thereby embodied in the capabilities and activities of the whole body. Moreover, we are also embodied persons for each other: we do not perceive a body-object whose movements lead us to infer an ‘inhabitant’ hidden in the brain like in a capsule. Rather, the lived body itself is the living appearance and expression of the person ‘in the flesh’.

## Body memory

As we have seen, the assurance of being-with-oneself, which the Cartesian subject believed to have found in self-observation and memory, always precedes these reflective acts. Now, one could object that this pre-reflective self-awareness only concerns a “minimal self” (Zahavi 2006) which barely satisfies our expectations of individuality and personhood. Bodily existence would give us just an anonymous identity or sameness, but no *qualitative identity*—being the sort of persons that we are. In order to adequately evaluate the significance of pre-reflective self-awareness for personal continuity, we have to further explore the *temporality* of this layer of experience.

First, we are dealing here not with an instantaneous, but with an extended and continuous self-experience, which is based on the unfolding and connection of protentions, impressions and retentions as analyzed by Husserl (1966). It is this connection which establishes the ongoing continuity of the pre-reflective self (Zahavi 1999, p. 73). This continuity is not restricted to fleeting experiences, however. It even extends over the entire life span once we consider the *history of the lived body*, which, in the course of a biography, becomes ever more a medium of our individual existence. All performances of life enter into the memory of the body and remain preserved as dispositions and potentialities: as Merleau-Ponty pointed out, the body is “solidified existence” and, for its part, “existence [is] perpetual incarnation” (1962, p. 148). Let us consider this history of the body in more detail.

The explicit or autobiographical memory with which Locke was concerned is by no means the only form of continuity which establishes itself over the course of our life. The majority of that which we have experienced and learned does not become accessible to us in retrospect, but far more in the practical movements of everyday life: habits are built up through repetition and practice and they are activated by themselves; established processes of movement are merged “into our flesh and blood”—such as walking upright, speaking or writing, dealing with instruments such as a bicycle or a piano. We can designate the totality of sedimented experiences as *implicit* or *bodily memory*. Already conceived in the work of Maine de Biran (1953) and Henri Bergson (1991), body memory envisions the past not in retrospect, but contains it as accumulated and currently effective experience. It is actualized through the medium of the body without requiring us to recall earlier situations (Schacter 1987; Fuchs 2008, 2012), or in other words, it is our *lived past*.

This implicit memory emerges in different types (Fuchs 2012), four of which I wish to sketch briefly:

1. As *procedural memory*, we may designate the already mentioned *sensorimotor* capabilities of the body: well-practiced habits, the skillful handling of instruments as well as familiarity with patterns of perception, acquired through repetition and practice. This memory relieves our attention from an overflow of details and enables the unreflective activities of everyday life. It facilitates the kind of action in which we turn our attention to the goal of the performance, rather than each individual movement; for example, the melody we wish to play with an instrument, and not the separate movements of our fingers.
2. *Situational body memory* enables us to recognize familiar situations and to skillfully cope with them. This concerns in particular spatial situations in which we find our bearings, such as in an apartment, in a neighborhood, or in a hometown. Bodily experiences connect particularly to interior spaces, and the more often this happens, the more the room is filled with a familiar, intimate atmosphere. ‘Inhabiting’ and ‘habit’ are equally grounded in body memory. The following example of Gaston Bachelard demonstrates this:
  But over and beyond our memories, the house we were born in is physically inscribed in us. It is a group of organic habits. After twenty years, in spite of all the other anonymous stairways; we would recapture the reflexes of the ‘first stairway’, we would not stumble on that rather high step. The house’s entire being would open up, faithful to our own being (…) The word habit is too worn a word to express this passionate liaison of our bodies, which do not forget, with an unforgettable house” (Bachelard 1964, 92f.).
  In this way, through its situational memory, the body connects with complementary environmental affordances (Gibson 1979) or “offerings”—things being graspable, viable, attractive, repulsive, etc., in accordance with the experiences and skills acquired before.
3. Intuitive, non-verbal communication with others, including the empathic understanding of their expressions, is also based on acquired capacities of the body, namely on the *intercorporeal memory*, which goes back as far as earliest childhood. In the first year of life, infants already learn patterns of social interactions with others which imprint themselves in their body long before the development of biographical memory, which occurs in the second year of life. In infant research, one speaks of *implicit relational knowledge* (Stern 1998)—how one shares pleasure with others, shows joy, avoids rejection, and so forth. We find another form of intercorporeal memory in well practiced dance partners who move easily with the rhythm of the music, and their hands and bodies interact without the need of any verbal or visual guidance.
4. Finally, body memory also includes those individual habits, attitudes and roles that have often been taken over from others and have been incorporated as an embodied personality structure; I have termed it *incorporative memory* (Fuchs 2006, 2012). For instance, the submissive behavior of an insecure person, his compliance and anxiousness, belong to a unified pattern of behavior and expression which was acquired in early childhood and now constitutes his personality. Thus, body memory also becomes the carrier of what has been called the *habitus* in sociology (Bourdieu 1990). It may be understood as a set of dispositions, skills, styles, tastes, and ways of acting, which are taken for granted or “go without saying,” and which are acquired through the social activities of everyday life. In this way, forms of cultural and class-specific socialization are integrated into the body memory and the manners of a person. “The habitus—embodied history, internalized as a second nature and so forgotten as history—is the active presence of the whole past of which it is the product” (Bourdieu 1990, p. 56).

We see how the continuous embodiment of existence produces a form of memory which from birth on integrates a person’s past into her present bodily constitution. Far from ensuring solely an anonymous, pre-reflective existence, the habitual body always forms an excerpt of personal history. It is the expression of our individuality at all levels, not just in the sophisticated ways of self-reflective thought, autobiographical memory or verbal interaction. This complies with Merleau-Ponty’s conception of the continuity of the bodily subject:

…so I am not myself a succession of ‘psychic’ acts, nor for that matter a nuclear I who brings them together into a synthetic unity, but one single experience inseparable from itself, one single ‘living cohesion’, one single temporality which is engaged, from birth, in making itself progressively explicit, and in confirming that cohesion in each successive present (1962, p. 363).

The rational and cognitive understanding of the person binds the conditions for personhood to intentional, conscious acts of remembering. A patient with high-grade dementia would then no longer be a person as he would no longer be able to remember his earlier states of being, maybe not even his name. However, this understanding of person separates selfhood from the body. The foundational continuity of a person does not depend on a stock of explicit knowledge and memories or his own biography. It depends, on the one hand, on the subject’s bodily self-familiarity: the pre-reflective awareness of self that never fully leaves us. And it is based furthermore on body memory, in other words, on *a history accumulated, sedimented in the body and, as such, implicitly always present.*

## Dementia and personal identity

Now we have prepared the ground for a deeper analysis of dementia. How should we conceive and evaluate the identity of demented persons, particularly in later stages of the illness? In what follows, I will develop a phenomenological account of dementia, which starts from the fundamental loss of reflexivity in dementia patients and then emphasizes the role of body memory for their personal continuity. Finally, I will take a critical look at social constructivist and narrative approaches to the problem of identity in dementia.

### Dementia as a loss of reflexivity and meta-perspective

Since the impairment of short-term memory and temporal orientation belongs to the earliest and most conspicuous symptoms of dementia, it is often regarded as the central disturbance of the disorder. However important though this progressive loss of memory may be, the more significant disturbance of dementia only shows in its further course. It is a fundamental impairment of the higher-order capacity of *reflexivity and decentering*, which was already pointed out by Zutt (1963), Tatossian (1987) and Summa (2014). What is at stake is the particular human capacity of stepping out of one’s bodily center and taking a virtual perspective on oneself, which is simultaneously the possible perspective of others – that what Plessner (1928/2019) has termed the “excentric position” of the human being. This crucial function includes a number of interrelated capacities such as the following:

- *Spatial and temporal orientation:* This is the capacity to rise above the immediacy of current experience in order to locate oneself in an objective geographical and temporal context. This requires mediating one’s own bodily orientation in the environment and day time – in the here and now – with the abstract orientation in objective space and time. Such mediation of two frames of reference becomes particularly manifest in using a map or a calendar.
- *Explicit recollection:* Autobiographical memory also requires stepping out of the ongoing current of implicit time (Fuchs 2017b) and to “re-present” a past experience, while being conscious of this stepping out and being able to at least approximately localize the experience within a calendrical time grid. Each dating of events implies stepping out of the immediate stream of time.
- *Reflection and self-distancing*: This includes capacities such as reflective thought, taking a stance toward oneself and the situation, anticipation and deliberation of future projects, social perspective-taking and moral judgment.
- *Symbolic or ‘as-if’ function*: This implies the distinction between reality and virtuality, the understanding of metaphors or proverbs, and the capacity of pretending or role-playing, capacities which again require a shifting between two frames of reference (Fuchs 2017c).

Dementia now means, at its core, a disturbance of these reflective and decentering functions, or to take another term, a loss of *meta-perspective*. This manifests itself in various phenomena, of which I only mention a selection:

- *Disturbances of spatial and temporal orientation* are a major characteristic of dementia, due to an inability to step out of the here and now. This amounts to an *enclosure in the present situation*, which can no longer be represented or seen “from above”; hence, maps and calendars become useless. With the loss of overview, the patients’ lived space narrows down to the immediate environment, beyond which, already in the imagination, there arises the threat of emptiness, disorientation and confusion, causing an elementary anxiety.
- *Explicit recollection* is not only impaired because of lost memory contents, but also because the chronological dating of experiences from a superordinate perspectives fails. Erratic memories pop up in one’s mind without clear localization in time. The loss of metaperspective and, thus, of a calendrical time grid leads to a growing fragmentation of the biographical structure of one’s life. In later stages of the illness the intentional awareness of remembering itself gets lost. This can result in an unnoticed “shifting” into an earlier phase of life – mistaking one’s wife for one’s mother, or one’s daughter for one’s sister, searching for one’s childhood home, etc. Frequently, different phases of one’s life overlap or even co-exist simultaneously.
- Among the various *disturbances of reflexivity and self-distancing* I only mention the impairment of perspective-taking (Theory of Mind) and of moral judgment, often leading to disinhibited behavior. This concerns frontotemporal dementia more than Alzheimer’s disease, however (Gregory et al. 2002; Lough et al. 2006; Bora et al. 2015). Perseverations and repetitions, which are frequently found in dementia, also indicate an inability to take a distance from a current behavior or situation.
- *Disturbances of the symbolic or ‘as-if’ function* refer to a failure of understanding non-literal language (proverbs, metaphors, irony; cf. Rapp and Wild 2011), but also in a missing distinction of reality and virtuality. Thus, in later stages dementia patients frequently mistake persons in the TV for real and may try to interact with them. In all these cases, the shifting between two different frames of reference fails, because the required superordinate perspective or ‘excentric position’ is missing.^8^

If we summarize these disturbance of reflexivity and meta-perspective, we can conceive of dementia as a *fundamental disorder of higher-order consciousness,* which must also lead to a fragmentation of the continuity of the reflective or narrative self. In this sense, the central disturbance in dementia concerns indeed what Locke considered a person to be, namely.

… a thinking, intelligent being, that has reason and reflection, and can consider itself as itself, the same thinking thing, in different times and places; which it does only by that consciousness which is inseparable from thinking. (Locke 1997, II.xxvii).

This is precisely what patients with dementia are no longer capable of: to reflect on their own existence, to identify certain past activities as ‘mine’, and to anticipate certain future situations as involving ‘me’ (Matthews 2006). What the patients lack, then, is *reflective self-consciousness,* or the capacity to “consider themselves as themselves”. However, as we have seen, the continuity of the person is by no means dependent on reflective or autobiographical continuity; rather, it is fundamentally based on her embodied, pre-reflective self. We will now take a closer look at this kind of continuity.

### Body memory in dementia

If dementia can be properly considered as a disturbance of the reflective layers of the self, then the pre-reflective levels become all the more important. The capacity to virtually step out of the situation is lost, but the patients still experience the world from their bodily center, and the immediate bodily connection to the environment is maintained. To be embodied means to be situated and oriented towards a field of experience as *this* body, as *this* history, *this* point of view (Bullington 2009); and this unique personal orientation conveyed by the lived body still exists. Hence, the patients will implicitly seek stability in a safe and familiar milieu which provides coherence and shelter against the disruption of reflective orientation in space and time. In contact with others, bodily modes of expression and behavior become more important than cognitive powers and the mostly diminished or fragmented speech acts. The more the patient’s behavior may be guided by familiar routines and environmental affordances, the less reflection and meta-perspective is required. This overall shift from abstract orientation, reflection and symbolic interaction to implicit habits and non-verbal capacities is based on the memory of the body (Fuchs 2008; Summa 2011).

While the progressive loss of explicit (autobiographical and semantic) memory is one of the earliest and most prominent symptoms of Alzheimer’s disease, vast ranges of implicit memory remain unimpaired even in the late stages of the illness. Accordingly, we can find well retained abilities in all of the above described forms of body memory. The realization of these abilities is, of course, bound to appropriate, complementary conditions of the surroundings. Let us look at the different types of body memory in more detail.

1. *Procedural memory:* Recognizing familiar faces and dealing with objects which provide affordances for a certain usage (cutlery, a toothbrush, or such like) remains possible in dementia for a long time, even if the names of persons or objects and their functions can no longer be designated. This corresponds to a *dissociation of explicit and implicit memory*, which is found in most of the forms of body memory as a result of the illness. Thus, procedural learning is still possible despite missing recollection; this can be verified by effects of priming or by motor or visual learning tasks (for example maze learning, mirror reading, etc.) – effects which are still comparable to those of healthy elderly people although the patients have no explicit memory of the tasks.^9^ Even learning to dance a waltz or the acquisition of other such skills are still possible.^10^ Musicians suffering from Alzheimer’s disease keep their skills for a long time, often even being able to learn new pieces. Generally, implicit musical memory (for example developing a liking for melodies heard repeatedly) is preserved much longer than explicit memory for melodies (Baird and Samson 2009).
2. *Situational memory:* As we have seen, body memory conveys the familiarity with situations—surroundings, voices, sounds and scents with their connotations and atmospheres. This situational memory is also effective in dementia. Instead of the reflective orientation in space and time, bodily orientation follows the primary directions and relations which the body establishes to the surrounding world, in accordance with the affordances of things: a chair serves ‘to sit upon’, a threshold ‘to cross’, a bed ‘to rest’, and so forth. The bodily habits and dispositions of being in the world can provide patients with elements of security and support. One of the most important tasks in support and care lies, therefore, in the maintenance of an appropriate spatial environment, preferably, of course, their own living space. But care homes can also establish personal spaces which convey at atmosphere of security.
  The utmost detailed knowledge of a patient’s biography, personal inclinations, and habits allows relatives and carers to bring about continuity and familiarity in the patient’s life. Known walking routes or locations often have a calming and stabilizing effect, even if the patient no longer has recognizable memories about them at their disposal. Certain sensory stimuli, in particular familiar music, can awake atmospheres, feelings, and capabilities, which are bound to past stages of life, though the memories relating to them have already faded away (Sung and Chang 2005). They may also elicit associated autobiographical memories which otherwise escape immediate grasp—as famously described in Proust’s experience of the *madeleine*, the tea-soaked biscuit which evokes the memories of his childhood. Accordingly, body memory may be therapeutically used in manifold ways, for example in art therapy, massage, music, rhythm and dance therapy, animal-based therapy, etc.^11^
  On the other hand, the dissociation of implicit and explicit memory in dementia may also lead to seemingly unmotivated emotions such as sadness, anger or anxiety, for which the patient cannot name a reason. For example, having experienced a distressing situation, the patient might sometime later, triggered by a similar object, person or event, become angry or begin to cry—for “no apparent reason” (Sabat 2006). Without an understanding of implicit memory, the person with dementia may then easily be considered as “irrationally hostile”, “emotionally labile”, or the like. Hence, careful observation and knowledge about the patients’ former experiences may help to better understand their reactions.
3. *Incorporative memory:* Body memory also contains the sedimented practices and habits which form a person’s character and role identity. Hence, patients with dementia usually show routines and patterns of behavior which correspond to their acquired habitus, though they may no longer fit to the current situation. For instance, a patient who has formerly worked as a nurse may mistake the care home for her work place, trying to take care of the laundry and giving instructions to other patients. Another patient known for her sense of politeness and hospitality might take great pains to set the table even if no guests are present. For those acquainted with her biography, this characteristic is a part of what makes her the person she is, a surviving fragment of a once much richer identity. Another example may illustrate the effect of once incorporated practices:
  A 78-year-old patient in an advanced stage of dementia was mostly incapable of recognizing his surroundings and his relatives any longer. He seemed lethargic, withdrawn, physically frail and was hardly in a position to move about independently any more. One day his two grandchildren visited him and were playing football in front of the house. As a youngster, the patient had played for a long while in a football club; now, he suddenly stood up and played with the boys. In contact with the ball, he appeared as if transformed and much younger; he showed them his skills at dribbling, demonstrated various ball tricks and gave expert explanations about these. For half an hour, almost nothing was discernible of the illness (case example from my own practice).
  The continuity of basal bodily self-experience in dementia is impressively demonstrated in such implicit actualizations of the life story (see also Kontos and Naglie 2009). The once-acquired habitus, such as a professional or sporting activity, is recalled by the relevant situation and its affordances, without requiring a biographical memory or explicit coordination.
4. *Intercorporeal memory:* Admittedly, even the procedural capabilities of body memory are not resistant against the illness in the long run. In the late stages of the process, patients lose not only biographical memories, but also, in the so-called *apraxia,* everyday skills, so that even a telephone or a toothbrush can become enigmatic objects. All the more, alongside the sensory-spatial and practical dimensions of body memory, *intercorporeality* represents the most important source of sustained continuity. The loss of verbal-cognitive powers means that non-verbal, emotional and bodily communication, as well as the *knowing how* of everyday modes of behavior, become ever more meaningful. Even in the advanced stages of the illness, the mimetic and gestural expressions of the patient can serve to give a differentiated disclosure of their condition and wishes (Hubbard et al. 2002; Becker et al. 2006; Kruse 2008).

Similarly, sufferers from dementia are particularly sensitive to the affective and atmospheric dimensions of contact. They are disposed to a differentiated world of feelings, of humor and, from time to time, surprising quick-wittedness, and, last but not least, a strong capacity for social bonding. They try to read the state of a relation (acceptance, evaluation, nearness or distance, etc.) from the intonation, facial expression and gestures of others and react sensitively to social atmospheres, for example to subtle signs of criticism or rejection. The interactions of patients are thereby less determined by conscious reflection or explicit attention to external norms than by the self-evident, pre-reflective nature of their embodied social habitus. Such repertoires of behavior are unjustly discredited as only “sustaining facades”. Rather, familiar modes of behavior allow the patients to establish affective relations with others and to fall back on basal intercorporeal orientation in situations which are rationally incomprehensible. It is, at the same time, their way of realizing their selves and confirming their existence as a person.

Precisely this necessity of self-confirmation demonstrates once again how the experience of self is maintained even in advanced dementia. What the patients lose is *reflexivity*, that higher-level capability of taking a stance toward one’s own experience or a momentary situation. Yet the pre-reflective self is not affected by this: indeed the patients experience their bodily here-and-now as well as their being-with others, above all, from an emotional point of view (Summa 2011). This manifests itself, for example, in the shame they feel with regard to failure or incapability, or in the exposure of the body before others; it also shows itself in their feelings of pride or joy in success or appreciation. Last but not least this can be seen in the case of conflicts which arise when the patient is minded to raise borders up around their personal space or to use force to articulate their wishes.^12^ The continuity of the basal and thoroughly personally-shaped feeling of self should thus prevent us from speaking of a loss of self in dementia.

### Relational versus embodied view of the person in dementia

The cognitive and individualistic concept of the self, which we have traced back to Descartes and Locke in the second section, has also been criticized from other sides, namely by representatives of social-constructivist or narrativistic views of the person (Kitwood 1997; Sabat and Harré 1992; Radden and Fordyce 2006). For them, personhood is bound to social relations and the attributions and the forms of recognition that result therefrom. The self or the personal identity of demented patients, so argue these authors, is retained in the recognition others show towards them, and in the narrative projections of the patient’s identity which others provide in their place. Thus, Kitwood defines the notion of the person as “the standing or status that is bestowed upon one human being by others, in the context of relationship and social being” (Kitwood 1997, p. 8), and concludes:

In dementia many aspects of the psyche that had, for a long time, been individual and ‘internal’, are again made over to the interpersonal milieu. Memory may have faded, but something of the past is known; identity remains intact, because others hold it in place; thoughts may have disappeared, but there are still interpersonal processes (Kitwood 1997, p. 69).

On the basis of a narrative concept of identity, Radden and Fordyce take a similar position:

The very self-awareness required to possess an identity depends upon and grows out of the contribution, and particularly the recognition, of other persons (Radden and Fordyce 2006, p. 72).

Finally, arguing for a social constructivist view of the self, Sabat and Harrè regard the Alzheimer disease sufferer as a “semiotic subject”:

Personhood can be an interpersonal discursive construction, a property of conversations […] The mind is no more than, but no less than, a privatized part of the ‘general conversation’ (Sabat and Harré 1994, 145f.)

The view that the self is mainly articulated through language and social relations has also determined the majority of studies on evidence of the persistence of self in dementia: they have usually focused on discourse, language, and narrative (Saunders 1998; Sabat 2002; Beard 2004; Addis and Tippett 2004; Surr 2006; Fazio and Mitchell 2009), whereas only one study investigated embodied selfhood in dementia (Kontos 2003, 2004; see Caddell and Clare 2010 for an overview).

Yet, as meaningful as intersubjectivity for the concept of person is, without a foundation in the subjectivity of the patient themselves, such attributions, narrative substitutes, or representations of interest by proxy are without adequate basis. Clearly, these have their significance for person-centered care, but they are first borne and supported by the modes of expression and behavior in which the personal identity and selfhood of the patients manifest right up to the end. Hence, even granted that the personal self is also relational in nature, such relations are always directed towards others as embodied subjects, and not as mere intersections of relations or projection surfaces.

Founding the self in dementia on an externalist or discursive account misses the 1st person perspective or the patient’s primary self-awareness in favor of a problematic social constructivist model of the self. As we have seen, an embodied view of dementia also emphasizes the implicit relations which the lived body establishes to the environment, and the ecological niche which conveys a sense of familiarity and belonging to the patient. However, it regards body memory, intercorporeality and interaffectivity as the basis of any person-centered approach to dementia, particularly in later phases of the illness. In contrast, narrative and constructivist views run the risk to ‘construe’ the patient instead of carefully perceiving his maintained personality in his bodily expressions and behavior. Moreover, they reach their limits when the patient’s capacity of verbal interaction subsides, whereas the embodied self is still attainable and tangible even in the last stages of dementia.

An embodied concept of the person, on the other hand, is based on a double foundation: on the one hand, on the continuity of organic life, which the body establishes even through phases of unconsciousness; on the other hand, on bodily subjectivity, which maintains a continuity of selfhood through body memory even when the ability of self-knowledge has been lost (Fuchs 2017b). The persistence of the person is therefore valid both from a 3rd person point of view, related to the organic body (*Körper*), and from the experience of the 1st person, related to the lived body (*Leib*). Personal existence means primarily bodily selfhood and being alive, from the beginning to the end. Reflective self-awareness, rationality and autonomy are, to choose a comparison, like the fruit on a tree, namely the appearance and product of its entire life, and cannot be separated from its bearer. They are abilities that are always actualized only for certain periods of time, but which are not sufficient to ensure the continuity of the person as such. Moreover, making them the sole condition for the attribution of personal status, as Peter Singer and other ethicists advocate, excludes a wide circle of people from being persons: newborns, severely retarded people and people with advanced dementia.

This is not intended to deny the importance of autobiographically and narratively conveyed identity. Amnesia and dementia diseases show sufficiently that the loss of explicit memory and autobiographical knowledge also fundamentally questions one's own selfhood. Conversely, however, self-reflection, memory and narration must be embedded in basal bodily self-acquaintance in order to constitute personal identity. In this respect, implicit and explicit, bodily and autobiographical memory contribute equally to personal identity. The loss of higher cognitive and self-reflective abilities such as self-knowledge or autonomous decision-making undoubtedly means a massive impairment of characteristics that we attribute to a healthy, adult person. All the more important are the interpersonal and relational forms of personhood and recognition that are able to replace those lost abilities to a certain extent. But this does not mean that the concept of person dissolves into mere relationality: all interpersonal relationships remain bound to the bodily appearance and continuity of the person.

## Conclusion

The concept of embodied personhood and history is able to change our image of dementia. In the place of a brain- and cognition-centered perspective, we may adopt the view of the patients in their own individual embodiment which, for its part, is embedded in social and environmental contexts. Even when dementia robs the patients of their explicit memories, they still retain their body memory, that means, their familiarity with environments, habits, sensory and motor memories. And instead of relying merely on rationality and autonomy, their self is seen as being primarily based on intercorporeality and interaffectivity, which remain in place despite the progress of the illness.

There is no question that higher-order capacities such as reflective thought and autobiographical memory are crucial for our everyday sense of identity—knowing what persons we are. In that sense people with severe dementia have admittedly lost part of their identity as persons. However, the memory of the body compromises a different, submerged history of the self. Its temporality does not follow the linear progress of the autobiographical life story, upon which we can purposefully retrieve. In body memory, the past continues much rather as organically accumulated and sedimented history, and it becomes effective in our personal forms of perception, behavior and interaction, without our being conscious of their particular origins. It is on this continuity and memory of the lived body that the identity of the patient is still founded.

Without doubt, the capacities and habits mediated by the body are part of the patients’ individual history, even though they may not be able to remember having learnt those capacities. Contrary to Locke’s view, their identity reaches further back than their explicit recollection. In our habitus, in our bodily being we manifest ourselves as persons no less than in our cognitive and reflective powers. If we understand selfhood as primarily bodily, we therefore arrive at a different perception of the patient with dementia: no longer as a person whom rationality and personality have abandoned, but rather as a person whose personhood can be precisely realized as both bodily and intercorporeal, so long as they can continue to live in the appropriate spatial, atmospheric and social surroundings. Their selfhood is preserved in the affinity of their body to the natural and social environment to which they have become accustomed. Their brains still contain “open loops”, that means, neuronal dispositions appropriate for interacting with the environment, which may be complemented by adequate affordances and offerings (Fuchs 2018). If this is provided, a coherent experience may still result despite cognitive and memory impairment, mediated through the pre-reflective familiarity of the body with the world.

A concept of person grounded solely in rationality and reflection inevitably stigmatizes people with severe cognitive deficits. On the other hand, in the framework of a concept of person orientated towards embodiment and intercorporeality, the response and relational capabilities of patients become a significant foundation of their personhood—such as the ability to give expression to joy, gratefulness, sorrow or fear that is still preserved. This elementary intercorporeal expressivity and responsiveness is the basis of the claim for respect, recognition and dignity which also persons suffering from dementia raise towards others. Their individuality remains present in the familiar sights, smells and melodies, in their dealing with things and their familiarity with other people – even though they are no longer able to account for the origin of this familiarity, nor to tell their life story. This is because the fundamental continuity of the person exists in the unified connection of their life, in the uninterrupted temporality of their body.

## Footnotes

1 Discussing the moral challenge of Alzheimer’s disease, Post (1995) has argued that Western cultures are ‘hypercognitive’, prizing cognition to the detriment of other aspects of being human which are no longer considered equally valuable.

2 For a critical discussion of McMahan’s position see Kittay (2008).

3 Cf. Kontos (2004), Fleischman et al. (2005), Golby et al. (2005), Harrison et al. (2007).

4 Principal representatives are, for example, Garrett (1998), Lewis (1976), Parfit (1984) or Shoemaker (1970).

5 I have investigated the continuity of the bodily self during states of deep sleep more extensively in Fuchs (2017b).

6 „There is, therefore, another subject beneath me, for whom a world exists before I am here, and who marks out my place in it. This captive or natural spirit is my body” (Merleau-Ponty 1962, p. 228).—“I am my body, at least wholly to the extent that I possess experience, and yet at the same time my body is as it were a ‘natural’ subject, a provisional sketch of my total being (p. 178).

7 This is because in order to identify a memory as an experience *I had*, I have to know myself already. If memory thus presupposes self-awareness or self-familiarity, it cannot ground my identity or continuity over time. This is equivalent to the objection already raised by Bishop Joseph Butler against Locke’s account of personal continuity: “… one should really think it obvious, that consciousness of personal identity presupposes, and therefore cannot constitute personal identity” (Butler 1736/1877).

8 The clock-drawing test, a well-known screening test for dementia, is also based on this failure of meta-perspective: A particularly sensitive component of the test is the drawing of the hands (usually the patient is asked to draw the date ‘10 min after 11’), for this implies the capacity to distinguish and shift between two different frames of reference – the digits meaning minutes or hours, respectively.

9 See Rösler et al. (2002), Eldridge et al. (2002), Fleischman et al. (2005), Harrison et al. (2007), and others.

10 These retained processes of motor learning correspond to the primary cortical localisation of most forms of dementia. Procedural and other forms of body memory are embedded predominantly in *subcortical* areas of the brain (basal ganglia, cerebellum, amygdala, amongst others) and, for a long period of the illness, remain there unimpaired (Schacter 1992; Squire 2004).

11 On this, see Filan and Llewellyn-Jones (2006), Guetin et al. (2009), Chancellor et al. (2014), Karkou and Meekums (2017).

12 A further indication of the retained feeling of self is shown in the competent use of the first-person pronoun as well as in self-reference in speech, mimic, and gesture. This can also be seen in the late stages of the illness (Fazio and Mitchell 2009).
